# Olfactory Dysfunction in Chronic Rhinosinusitis: Mechanisms, Diagnosis, and the Role of Endoscopic Sinus Surgery

**DOI:** 10.3390/jcm15124797

**Published:** 2026-06-20

**Authors:** Nikolaos Tsetsos

**Affiliations:** Department of Otorhinolaryngology-Head and Neck Surgery, 424 Military Training Hospital of Thessaloniki, 56429 Thessaloniki, Greece; tsetsosnikos@yahoo.gr

**Keywords:** chronic rhinosinusitis, olfactory dysfunction, smell loss, endoscopic sinus surgery, functional endoscopic sinus surgery (FESS), nasal polyps, type-2 inflammation, eosinophilia, olfactory epithelium, psychophysical testing, intranasal corticosteroids, biologic therapy, olfactory training

## Abstract

Chronic rhinosinusitis (CRS) constitutes a multicausal inflammatory disease of the nose and paranasal sinuses, often associated with olfactory dysfunction (OD), a symptom that significantly impacts patients’ quality of life. OD in CRS was traditionally thought to be related to mechanical obstruction of the olfactory cleft, but is now considered to be multifactorial, involving conductive, inflammatory, and sensorineural mechanisms as well. Type-2 inflammatory response (high interleukins IL-4, IL-5, IL-13), eosinophilia, and increased IgE are involved in epithelial damage, impaired neurogenesis, and persistent olfactory loss, especially in chronic rhinosinusitis with nasal polyps (CRSwNP). In addition, peripheral chronic inflammation may also play a role in central neural remodeling, which may potentially affect olfactory function. Objective psychophysical testing is necessary to accurately assess olfactory function because self-reports may lack reliability. Management strategies aim at reducing inflammation and restoring sinonasal ventilation. First-line therapy with intranasal corticosteroids and short courses of systemic corticosteroids may be useful for symptomatic relief. Biologic agents directed against type-2 inflammation have demonstrated significant benefits in selected cases. Functional Endoscopic Sinus Surgery (FESS) plays an important role in the treatment of refractory CRS to restore the airflow and to improve the delivery of topical drugs. Olfactory outcomes following surgery, however, are variable and often incomplete, reflecting underlying inflammation and neuroepithelial damage. Disease recurrence, especially in type-2–driven CRS, affects long-term outcomes, underscoring the necessity to incorporate surgery in an individualized, endotype-informed treatment strategy.

## 1. Introduction

Chronic rhinosinusitis (CRS) is defined as the inflammation of the nasal and paranasal sinuses characterized by symptoms lasting at least 12 weeks, including nasal obstruction, rhinorrhea, facial pressure, and reduction or loss of smell. It is typically classified into two subtypes, CRS with nasal polyps (CRSwNP) and CRS without nasal polyps (CRSsNP), based on the presence of nasal polyps, phenotypes that are characterized by different inflammatory pathways and response to available treatments [1,2,3].

Olfactory dysfunction (OD) is a prevailing symptom of CRS, especially in patients with the CRSwNP subtype [2,4]. It is well established that hyposmia or anosmia causes safety issues, nutrition problems, as well as social and emotional problems [4]. Although being one of the most common symptoms, olfaction is usually underestimated, as nasal blockage or discharge often takes priority in clinical evaluation [1,2,3,4,5].

Olfactory dysfunction in CRS patients has been traditionally attributed to obstruction of the olfactory cleft due to oedema and nasal polyps, supporting the conductive mechanism. However, over the last few years, there has been increasing evidence about other mechanisms, such as inflammatory and sensorineural ones. Type-2 inflammatory mediators and infiltration with eosinophils may cause damage to the olfactory epithelium, reduce neuroregenerative capacity, and compromise signal transmission [6,7]. This may be an explanation why patients who undergo surgery continue to experience olfactory dysfunction, although the conductive element represented by the nasal polyps has been eliminated.

It has been widely accepted that although conventional treatment with intranasal corticosteroids, oral steroids, and functional endoscopic sinus surgery can improve olfactory function, the degree of improvement is variable and often incomplete. Novel treatment modalities such as monoclonal antibodies targeting type-2 inflammation have been introduced, showing favorable results; however, their long-term impact has not been established [3,5,8,9].

The aim of this review is to collect all existing evidence regarding the mechanisms underlying OD in CRS, (2) approaches to assessment and monitoring of olfactory function, and (3) the effects of available and emerging therapies on olfactory outcomes and the role of Functional Endoscopic Sinus Surgery (FESS).

Although recent reviews, guidelines, and position papers have addressed specific aspects of olfactory dysfunction in chronic rhinosinusitis, including inflammatory mechanisms, biologic therapies, and disease management, a comprehensive synthesis of pathophysiology, clinical assessment, biomarkers, medical therapies, olfactory training, and the evolving role of endoscopic sinus surgery is limited. The present review aims to provide an update and a clinically oriented overview of olfactory dysfunction in CRS, with particular emphasis on the interaction of conductive and sensorineural mechanisms and the role of endoscopic sinus surgery in modern multimodal and endotype-based treatment concepts.

## 2. Literature Search Strategy and Study Selection

This review was conducted as a narrative review and was not designed or reported as a formal systematic review according to the Preferred Reporting Items for Systematic Reviews and Meta-Analyses (PRISMA) guidelines. Nevertheless, a structured literature search and study selection process was performed to enhance transparency, reproducibility, and methodological rigor.

The inclusion and exclusion criteria were defined clearly and comprehensively prior to the review process, to minimize the risk of selection bias. The inclusion criteria were original research articles, including observational studies (cohort, case–control, cross-sectional), clinical trials, reviews, systematic reviews, and meta-analyses relevant to CRS-related olfactory dysfunction. Studies were excluded if they were non-English language papers, studies with incomplete data, case reports, editorials, commentaries, non-peer-reviewed articles, duplicates, unavailable full texts, or abstract-only papers.

A comprehensive literature search was conducted using the PubMed/MEDLINE, Scopus, and Google Scholar databases to identify studies relevant to olfactory dysfunction in chronic rhinosinusitis (CRS). The search included publications available up to 10 May 2026 using multiple combinations of the free-text terms “chronic rhinosinusitis”, “olfactory dysfunction”, “smell loss”, “anosmia”, “hyposmia”, “olfaction”, “nasal polyps”, “chronic rhinosinusitis with nasal polyps”, “chronic rhinosinusitis without nasal polyps”, “endoscopic sinus surgery”, “functional endoscopic sinus surgery”, “biologic therapy”, “dupilumab”, “type 2 inflammation”, “eosinophilic inflammation”, “olfactory training”, and “quality of life”.

The search yielded 854 records, including 348 records from PubMed/MEDLINE, 287 records from Scopus, and 219 records from Google Scholar. After the removal of 221 duplicates, 633 records underwent title and abstract screening. During this stage, 489 records were excluded because they were not directly related to CRS-associated olfactory dysfunction, focused on non-rhinologic causes of smell loss, represented conference abstracts, editorials, or letters without relevant original data, or lacked sufficient relevance to the objectives of the review.

Subsequently, a total of 144 articles were sought for retrieval and successfully obtained for full-text assessment. Full-text articles were considered eligible if they addressed one or more aspects of olfactory dysfunction in CRS, including epidemiology, pathophysiology, inflammatory mechanisms, clinical assessment, biomarkers, medical treatment, biologic therapies, olfactory rehabilitation, or surgical management. Systematic reviews, meta-analyses, randomized controlled trials, prospective and retrospective cohort studies, clinical practice guidelines, consensus statements, and landmark publications providing substantial contribution in the understanding of CRS-associated olfactory dysfunction were prioritized. Manual review of the reference lists of selected articles identified additional relevant studies.

Following full-text assessment, 81 articles were excluded. The primary reasons for exclusion included insufficient relevance to the objectives of the review (*n* = 39), non-English language publications (*n* = 12), conference abstracts, editorials, or publications without adequate primary data (*n* = 15), and insufficient clinically relevant information regarding olfactory dysfunction in CRS (*n* = 15).

**Figure 1:**
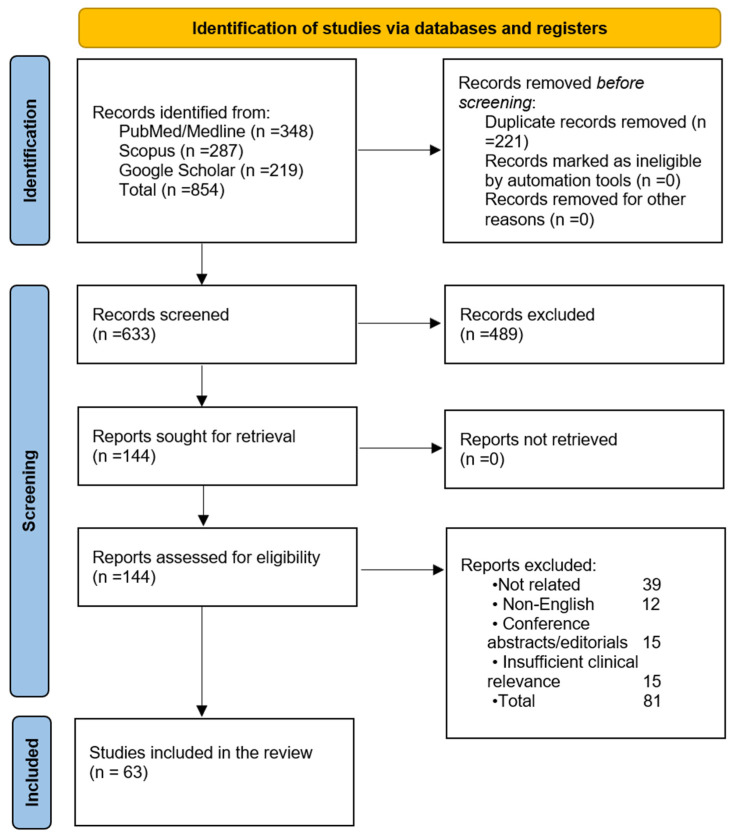
PRISMA flow diagram of the study selection process.

Finally, 63 studies were included in the narrative synthesis. Although this review was not performed according to formal PRISMA methodology, a PRISMA-style flowchart was included to improve transparency in the process of study identification and (Figure 1). Our review of published articles did not require informed consent or ethics approval.

## 3. Epidemiology

Olfactory dysfunction is a common complaint among patients with chronic rhinosinusitis, impairing significantly their overall quality of life. Literature shows that around 50–80% of patients with CRS demonstrate measurable impairment, with the highest levels reported in CRS patients with nasal polyposis [2,4,10]. The inflammatory burden, as indicated by the endoscopic and radiographic findings, may serve as a predictor of the olfactory function, highlighting the relationship between disease status and olfactory loss [3,10]. Surprisingly, even patients who do not complain about olfactory dysfunction often have psychophysical testing that reveals performance below normative standards, implying that self-reported symptoms do not fully capture the true burden [3,4].

## 4. Variation Across Phenotypes and Endotypes

It is well established that olfactory dysfunction varies across different CRS phenotypes and inflammatory endotypes. Patients with CRSwNP almost always exhibit more severe and persistent olfactory loss as compared with those with the CRSsNP subtype, regardless of the disease control [2,4]. Type-2 inflammation, which dominates the CRSwNP subtype, is characterized by high levels of interleukins (IL-4, IL-5, IL-13), tissue infiltration with eosinophils, and increased local IgE, all of which have been linked to epithelial damage and compromised olfactory neurogenesis [7,11,12].

Patients with comorbidities associated with type-2 inflammation, such as asthma, aspirin-exacerbated respiratory disease (AERD), are prone to experience profound and recurrent olfactory loss, rendering them independent factors of olfactory dysfunction in CRS patients [3,13,14]. Lastly, central compartment atopic disease (CCAD) constitutes another CRS subtype of type 2 CRS, defined as an inflammation and edematous change of the central sinonasal compartment, including the middle turbinate, superior turbinate, and posterosuperior nasal septum. It has been found that central-compartment-type CRS, represented by an eosinophilic/type 2 inflammation endotype, with high levels of IL-5 and IL-13 in the sinonasal mucosa, causes more severe olfactory dysfunction as compared with patients with other CRS subtypes [15,16].

## 5. Impact on Daily Life and Patient-Reported Outcomes

Olfactory dysfunction results in a significant and often underestimated effect on patients’ quality of life. Patients often report diminished gustatory pleasure, altered eating behavior, reduced ability to detect environmental hazards such as gas and smoke, and reduced attunement to environmental and social signals [4,5,8,16]. Psychological impact is also considerable. Olfactory dysfunction is also associated with higher rates of depression, anxiety, and social withdrawal, and has been linked to impairments in occupational and daily functioning that mirror those observed in comparable chronic diseases [5,10]. Greater social insecurity and a decreased sense of pleasure in shared eating can foster social withdrawal, a process that may be further intensified by concerns about one’s professional future. This decline in olfaction-related quality of life may, in turn, contribute to increased depressive symptoms. In addition, changes in brain function following olfactory loss may influence how emotions are processed [17].

## 6. Pathophysiology of Olfactory Dysfunction in CRS

### 6.1. Conductive Mechanisms

#### 6.1.1. Obstruction of the Olfactory Cleft

Impaired airflow to the olfactory cleft, which impedes odorants from reaching the olfactory cleft, has been established as the main mechanism causing olfactory dysfunction in CRS patients. Mucosal oedema, mucus retention, and structural narrowing of the superior nasal cavity decrease odorant contact with the epithelium, leading to reduced detection thresholds, although neural function may not have been impaired [2,10,17].

#### 6.1.2. Polyps, Edema, and Mucus

Nasal polyps covering the olfactory cleft exacerbate the conductive problem and disrupt normal airflow patterns. The presence of polyps induces turbulent airflow and shortens odorant exposure to the olfactory mucosa, ultimately impairing odorant transport. Inflammatory edema and thick mucus further worsen the effect by lining the olfactory mucosa and restricting odorant diffusion to receptor neurons. It is well established that olfactory improvement can be achieved by addressing olfactory cleft obstruction and restoring the airflow with conservative treatment (intranasal or oral steroids) or FESS [18,19,20].

### 6.2. Sensorineural Mechanisms

#### 6.2.1. Inflammatory Injury to the Olfactory Epithelium

Chronic rhinosinusitis causes direct inflammatory damage to the olfactory epithelium. Histopathologic changes shown by several studies constitute squamous metaplasia, loss of olfactory supporting cells, epithelial erosion, and a reduction in the number of mature olfactory sensory neurons compared with normal mucosa. These findings are associated with increased infiltration of inflammatory cells and disorganized epithelial structure, underscoring that rather than being limited to a simple conductive blockade, the nasal mucosa exhibits structural remodeling driven by ongoing inflammation [21,22].

Olfactory epithelium damage is influenced by a dominant type-2 inflammatory response characterized by elevated IL-4, IL-5, IL-13, and eosinophilia, which compromises epithelial barrier integrity, contributes to cytotoxic tissue damage, and is linked to diminished olfactory function and resistance to therapy [7,11]. Over time, chronic inflammation leads to impaired regeneration of the olfactory epithelium, diverting progenitor cells toward non-olfactory differentiation and leading to maladaptive remodeling that limits neuronal replacement. The combination of structural injury, inflammatory toxicity, and impaired regeneration may explain why olfactory dysfunction in CRS often persists despite successful restoration of sinonasal patency [7,23].

#### 6.2.2. Central Neural Contributions

Recent studies have shown that CRS can interfere with the central olfactory system, not just the peripheral nerves. Imaging studies using magnetic resonance imaging (MRI) have suggested that patients with CRS, particularly those with significant olfactory dysfunction, may exhibit reduced olfactory bulb volumes compared with healthy controls. In a prospective cohort study, olfactory bulb volumes were significantly lower in patients with nasal polyps than in matched controls, and these patients experienced an increase in their volumes combined with olfactory improvement following FESS [24]. Olfactory bulb atrophy on MR images was significantly higher in patients with olfactory dysfunction than in those with normosmia, as studied in patients with diverse causes of smell loss. Thus, assessing olfactory bulb atrophy on MRI may provide a practical, objective tool for verifying olfactory impairment in patients who report smell loss. These results reinforce the concept that chronic peripheral deficits can ultimately produce detectable structural alterations in central olfactory regions [25].

Although these findings support the concept of central neural involvement in CRS-associated olfactory dysfunction, the clinical significance of such imaging abnormalities remains incompletely understood [24,25,26]. Most of the available evidence stems from relatively small observational studies, and it remains uncertain whether these central changes are a cause of persistent olfactory dysfunction, a consequence of decreased sensory input, or a combination of both [24,25,26,27].

Beyond volumetric changes, CRS-related inflammation of the olfactory epithelium reduces afferent sensory input, which in turn causes alterations in central olfactory processing and neural plasticity. Experimental evidence further suggests that chronic peripheral inflammation may influence central olfactory pathways. Animal studies have shown that chronic inflammation of the nasal cavity leads to the activation of microglia and astrocytes in the olfactory bulb, indicating a possible association between peripheral inflammation and central olfactory remodeling. However, in human CRS, direct evidence of clinically relevant central inflammatory propagation is still limited [26,27]. Reduction of olfactory input leads to reorganization of synaptic networks and microglial-neuronal interactions, highlighting the ongoing experience-dependent plasticity of central olfactory pathways [27].

Taken together, these findings suggest that CRS-associated olfactory dysfunction involves abnormalities throughout the olfactory pathway, ranging from peripheral injury of the olfactory epithelium to structural and functional changes within central olfactory networks. This integrated model highlights the importance of considering both sensory input and neural plasticity when interpreting olfactory loss and treatment outcomes. Although current imaging studies provide valuable insights into the pathophysiology of CRS-related olfactory dysfunction, their clinical applicability remains limited by the predominantly observational nature of the available evidence and the relatively small study populations. Further studies are required to determine whether imaging markers of central olfactory remodeling can serve as reliable tools for prognosis, treatment stratification, or monitoring of therapeutic response [24,25,26,27] (Table 1).

## 7. Clinical Assessment of Olfactory Function

Detailed evaluation of olfactory function is cardinal in CRS patients as smell loss is very common, often progressive, and closely linked to reduced quality of life. Assessment with objective tools, therefore, assists in separating true olfactory loss from subjective perception, identifying potentially reversible etiologies, and monitoring responses to conservative and surgical treatment [7,10,28].

### 7.1. History and Symptom Questionnaires

A thorough medical history should address the onset of symptoms (sudden vs. gradual), triggering factors (e.g., viral illness), and pattern (stable, fluctuating, or progressive), as well as qualitative olfactory disorders such as parosmia and phantosmia, which gain recognition in sinonasal disease. Patient-reported outcome measures assess the perceived weight of symptoms and evaluate aspects that objective tools cannot, including enjoyment of food, safety issues, and emotional problems. Assessment tools such as the 22-item Sinonasal Outcome Test (SNOT-22) and smell-specific questionnaires are useful for monitoring change over time [2,10,29].

### 7.2. Objective Psychophysical Testing

Objective psychophysical tools are considered the gold standard for assessing olfactory function as they yield quantitative, reproducible data across different aspects of olfactory function [3,7,28]. These tools study odor threshold (sensitivity to odors), discrimination (ability to differentiate between odors), and identification (ability to correctly name or recognize odors). The University of Pennsylvania Smell Identification Test (UPSIT), a 40-item “scratch-and-sniff” identification test, is one of the most commonly used assessment tools, demonstrating high reliability and permitting classification of normosmia, hyposmia, and anosmia, making it useful for both clinical and research purposes [30].

The second most widely used assessment tool is the Sniffin’ Sticks battery, which evaluates threshold, discrimination, and identification using scented markers. The combined TDI (Threshold–Discrimination–Identification) score provides a broad clinical profile and is especially sensitive for detecting longitudinal change in CRS patients [31]. Shorter screening tools—including the Brief Smell Identification Test—may help triage patients but lack the precision needed for treatment decisions [32].

Self-reported olfactory ability is often unreliable. As many as one-quarter of patients with olfactory dysfunction are unaware of their deficit, likely reflecting the gradual decline in smell that accompanies the chronic course of CRS; therefore, psychophysical testing plays a crucial role in predicting outcomes after medical or surgical treatment, and documenting improvement following interventions such as corticosteroids, biologics, or FESS [28,33,34]. When possible, periodic assessments offer important information about disease progression and treatment response.

### 7.3. Imaging and Endoscopy

Nasal endoscopy is imperative in CRS patients as it allows direct visualization of the middle meatus and olfactory cleft for polyps, edema, crusting, discharge, scarring, or masses, especially at the olfactory cleft and the ostiomeatal complex. Endoscopic findings can be objectively quantified and recorded with the use of validated scoring systems, such as the Lund–Kennedy scale, which quantifies the presence and severity of nasal polyps, edema, and discharge, or the olfactory cleft endoscopy score [4,28,31,35]. Computed tomography (CT) imaging further assesses the extent of disease, reveals obstruction of the olfactory cleft, and helps guide pre-surgical planning. Radiologic evaluation of the paranasal sinuses and the ostiomeatal complex is typically carried out using the widely utilized Lund–Mackay scoring system, which grades the degree of sinus opacification and ostiomeatal complex involvement on CT imaging [4,28,35,36].

### 7.4. Biomarkers and Emerging Tools

In recent years, there has been increasing interest in identifying biomarkers that may help to understand the heterogeneity of olfactory dysfunction in CRS. Elevated type-2 inflammatory mediators, including IL-5, eosinophils, periostin, and IgE, have been associated with worse baseline olfactory function and an increased likelihood of persistent or recurrent disease, suggesting a potential role for inflammation-mediated sensorineural injury [11,12,37]. While these findings are promising, the clinical utility of any individual biomarker to predict olfactory outcome or inform treatment decisions is not yet fully established [37,38]. Biomarker-based approaches may have a role in treatment stratification in the future, particularly in relation to biologic therapies and endotype-driven management [11,37,38]. New research tools, such as olfactory event-related potentials, functional neuroimaging, and molecular profiling, offer valuable insights into the mechanisms of olfactory dysfunction [24,25,26,27]. These techniques, however, are still mainly investigational and require further validation before wide clinical use [24,25,26,27,38]. Currently, the main tools in routine clinical assessment are psychophysical olfactory testing, nasal endoscopy combined with imaging [3,38].

## 8. Management Strategies

It has been shown that olfactory dysfunction in CRS patients is reversible to a limited extent for many patients, and most treatments confer at least modest effects on olfactory function by reducing inflammation, promoting better nasal aeration, or targeting type-2 pathways. Corticosteroids (topical and systemic), FESS, and monoclonal antibodies have all been demonstrated to improve psychophysical test results and quality of life related to olfaction, although the extent and persistence of benefit differ among phenotypes/endotypes and disease severity [17,20,38].

### 8.1. Topical Therapies

Intranasal corticosteroids are considered first-line treatment for CRS and constitute the backbone of conservative management of CRS-associated olfactory loss as well. They act by reducing mucosal oedema, shrinking polyp burden, and modulating local type-2 inflammation, which enhances odorant access to the olfactory cleft while limiting inflammatory damage to the olfactory epithelium [17,38]. A Cochrane systematic review of randomized controlled trials with a total of 2738 participants with chronic rhinosinusitis found moderate-quality evidence that intranasal corticosteroids improve symptom scores, including loss of sense of smell, compared with placebo or no treatment [39]. A meta-analysis limited to CRSwNP reported that topical corticosteroids lead to significant improvements in olfaction, whether used alone or combined with systemic steroids [40].

As the efficacy of intranasal corticosteroids may vary according to the way of administration, newer delivery strategies (drops, irrigations, exhalation delivery systems, steroid-eluting implants, targeted deposition into the olfactory cleft) have been examined to enhance the delivery of medications to the olfactory cleft, however data are still emerging, and not all devices are widely available. Overall, intranasal corticosteroids are generally considered safe and widely available, and they should typically be maintained long term in CRS patients with olfactory impairment, with the understanding that benefit is often incomplete and varies according to disease endotype [17,38,40].

### 8.2. Systemic Corticosteroids

The effects on olfactory function of oral corticosteroids in patients with CRSwNP who present with increased type-2 inflammation may be rapid and even striking. Their primary therapeutic actions are a quick decrease in polyp load, mucosal oedema, and infiltration of eosinophils, which often leads to significant short-term improvement in olfactory function. In a double-blind, randomized study with patients with CRSwNP, oral prednisolone supplemented with topical corticosteroids gained significantly greater improvements in polyp size, nasal symptoms, and smell perception over 6 months, versus topical corticosteroids alone [41]. Analogously, in a case of a tapering dose of oral methylprednisolone and doxycycline and placebo in CRSwNP patients, the greatest and quickest reductions in polyp size and symptoms were observed for systemic corticosteroids, with olfactory function demonstrating the most marked improvement; however symptoms rapidly worsened after the last dose [42]. A Cochrane systematic review of short courses of systemic steroids in patients with CRS has reported that oral corticosteroids, whether used as monotherapy or adjunct to therapy, provide transient relief of symptoms and endoscopic findings, but evidence regarding sustained olfactory benefit over time is still limited [43].

Recovery of olfactory function is often only temporary, but most patients have ongoing olfactory loss when inflammation returns after the therapy is discontinued. Because systemic corticosteroids pose significant dose- and duration-dependent risks—including metabolic, skeletal, cardiovascular, and psychiatric effects—the European Position Paper on Rhinosinusitis and Nasal Polyps 2020 (EPOS 2020) recommends limiting their use to short “rescue” courses in selected cases [3]. These are usually patients with severe CRSwNP, with a high level of inflammation and a large amount of smell impairment, or who are in the pre-operative state. In the long-term, the focus should be on topical corticosteroids, and—when indicated—steroid-sparing biologics. If additional treatments are to be instituted further, the risks and benefits should be judged carefully by decision-makers, and the history of previous treatment response should be documented [3,43].

### 8.3. Role of Functional Endoscopic Sinus Surgery in Olfactory Dysfunction

#### 8.3.1. Restoration of Olfactory Cleft Ventilation

Functional Endoscopic Sinus Surgery (FESS) improves olfactory function mainly by restoring airflow to the olfactory cleft (conductive mechanism). In cases of CRS, mucosal edema, nasal polyps, and retained secretions block the access of odorants to the olfactory epithelium. The removal of these obstructive pathologies and the widening of the sinonasal pathways allows odorants to diffuse to the olfactory epithelium and enhances odor detection. Several studies have proved that removal of sinonasal obstruction improves odorant access to the olfactory epithelium and contributes to recovery of olfactory function in patients with chronic rhinosinusitis [5,44]. Clinically, improvement in olfactory function is usually observed within the first few months after surgery, particularly in patients with significant olfactory cleft obstruction caused by nasal polyps or mucosal edema. Previous studies have demonstrated that patients with baseline anosmia and nasal polyposis tend to experience greater postoperative olfactory improvement than those with milder olfactory impairment [45,46]. Nevertheless, postoperative olfactory outcomes are still inconsistent, suggesting that restoration of airflow alone does not fully explain recovery of smell function and that other inflammatory and neuroepithelial factors are involved in long-term outcomes [45,47].

#### 8.3.2. Interaction with Inflammatory Burden and Sensorineural Limitations

Although restoration of airflow is essential, olfactory function after FESS is strongly associated with underlying inflammatory and sensorineural factors. Chronic inflammation within the olfactory cleft may cause epithelial damage, neuronal loss, and impaired neurogenesis, which are only partially reversible with surgery [21,22,23]. Consequently, patients with severe type-2 inflammation, eosinophilia, or persistent inflammatory disease often experience incomplete recovery despite appropriate surgical management [11,12]. This highlights that olfactory dysfunction in CRS is not purely conductive, but is attributable to both structural obstruction and inflammation-induced neuroepithelial injury [47,48].

From a clinical perspective, olfactory improvement following FESS is most likely when obstruction of the olfactory cleft is the dominant mechanism of smell loss. In contrast, when chronic inflammation has already caused epithelial remodeling, loss of mature olfactory sensory neurons, or impaired regenerative capacity, surgery may restore ventilation and topical drug access but may not fully reverse olfactory dysfunction [21,22,23]. This distinction is important for preoperative counseling, because technically successful surgery may improve nasal obstruction and sinus ventilation while producing only partial or transient recovery of smell in patients with advanced inflammatory or neuroepithelial injury [47,48].

#### 8.3.3. Enhancement of Postoperative Medical Therapy

An important aspect of FESS, often overlooked, is its ability to enhance the delivery and therapeutic efficacy of topical therapies. FESS reestablishes patency of the sinus ostia and improves access to the olfactory cleft, allowing better intranasal drug deposition and irrigation delivery, which is crucial to control persistent inflammation. Improved delivery of topical therapies has been associated with improved long-term symptom relief and may play a role in sustained improvement in olfactory function when complemented by optimal post-operative medical therapy [49,50,51]. This concept is in line with the current perception of FESS as a facilitator of long-term disease control rather than a stand-alone procedure. Surgery may enhance the effectiveness of postoperative medical therapy and may assist in maintaining olfactory improvements following surgery by enhancing the availability of topical corticosteroids and saline irrigations to the sinonasal cavity and olfactory cleft. Thus, long-term improvement in smell function is generally dependent on not just surgical success but also compliance with postoperative anti-inflammatory therapy regimens [3,38,49,50,51].

#### 8.3.4. Predictors of Olfactory Improvement and Variability of Outcomes

Olfactory outcomes following FESS are heterogenous and various predictors of response have been identified. Better improvement is generally observed in patients with nasal polyps, greater olfactory dysfunction at baseline, and shorter disease duration. On the contrary, type-2 inflammation-related comorbidities, such as asthma and aspirin- exacerbated respiratory disease (AERD), are associated with poorer or less durable olfactory recovery. Preoperative psychophysical testing has also been shown to correlate with postoperative outcomes, supporting its role in patient selection and counseling [45,52,53,54].

Several studies indicate that patients with CRS with nasal polyps (CRSwNP) and significant olfactory cleft obstruction may derive the greatest olfactory benefit from surgery, particularly when smell loss is predominantly conductive in nature. Interestingly, patients with more severe baseline olfactory dysfunction tend to demonstrate larger postoperative gains, likely because relief of obstruction produces a greater relative improvement. On the other hand, patients with long-standing disease, recurrent polyps, severe eosinophilic inflammation, asthma, or aspirin-exacerbated respiratory disease (AERD) tend to have less predictable and permanent recovery. These findings underline the need for detailed preoperative counseling and highlight the need to evaluate both inflammatory burden and the integrity of the underlying neuroepithelium when predicting postoperative olfactory outcomes [13,14,45,53,54].

#### 8.3.5. Long-Term Outcomes and Role Within Multimodal Management

Although a substantial proportion of patients experience early improvement in olfactory function following FESS, long-term outcomes remain less predictable. Sustained olfactory benefit is closely linked to effective postoperative control of sinonasal inflammation. Recurrence of mucosal inflammation, polyp regrowth, and persistent type-2 inflammatory activity may lead to gradual deterioration of olfactory function over time, particularly in patients with nasal polyps. Therefore, FESS should not be regarded as a curative intervention but rather as one component of a comprehensive treatment strategy. Contemporary management of CRS-associated olfactory dysfunction increasingly relies on a multimodal approach that combines surgery with long-term topical corticosteroid therapy, saline irrigations, intermittent systemic treatment when indicated, and, in selected patients, biologic agents targeting type-2 inflammation. Such an integrated strategy is essential for maintaining olfactory improvement and achieving long-term control of disease progression [3,17,20,54,55,56].

### 8.4. Biologic Therapies

Monoclonal antibodies targeting type-2 inflammatory pathways significantly improved the management of severe chronic rhinosinusitis with nasal polyps (CRSwNP), with particularly notable benefits for olfactory function. In the phase 3 LIBERTY NP SINUS-24 and SINUS-52 studies, dupilumab—a monoclonal antibody targeting the IL-4 receptor alpha—showed marked, rapid, and sustained improvements in both self-reported smell and objective UPSIT scores compared with placebo, additionally reducing nasal polyp burden and sinus opacification [9,57].

Other monoclonal antibodies that target IgE or IL-5/IL-5R (e.g., omalizumab, mepolizumab, benralizumab) have also shown improvement in olfaction; however, effect sizes for psychophysical tests appear moderately reduced and less consistent compared with dupilumab [58,59]. Monoclonal antibodies in CRSwNP consistently offer clinically meaningful smell improvement, especially in patients with severe baseline olfactory dysfunction, extensive nasal polyposis, and a strong type-2 inflammatory profile. This fact makes these treatments especially appealing for patients who rely on systemic steroids, have failed sinus surgery, or cannot safely use systemic steroids; however, considerations such as high cost, availability, and the requirement for ongoing treatment need to be carefully balanced [5,7,9,58].

Although biologic therapies have shown meaningful improvements in olfactory outcomes in selected patients with type-2 CRS, important questions have been raised regarding optimal patient selection, duration of treatment, long-term efficacy, and cost-effectiveness. Furthermore, determinants of the olfactory response have not been well characterized. Therefore, biologics should be examined in the setting of the overall strategy of personalized and endotype-driven management of CRS rather than as a stand-alone treatment for olfactory dysfunction [9,57,58,59,60].

### 8.5. Adjunctive Approaches

Olfactory training, which entails planned and repeated exposure to specific odors over a course of several months (usually 4 different odors twice daily for at least 24 weeks), is a meaningful, low-risk, non-pharmacological and non-surgical treatment option that can be recommended for many patients with CRS-related olfactory dysfunction. Several randomized and prospective studies involving patients with olfactory dysfunction of different etiologies, including post-infectious, post-traumatic, and idiopathic smell loss, have demonstrated that OT may improve Sniffin’ Sticks measures of threshold, discrimination, and identification compared with no training, although the magnitude of benefit varies among individuals [60,61].

In CRS specifically, the available evidence remains relatively limited compared with other forms of olfactory dysfunction. Nevertheless, emerging data suggest that OT may be a valuable addition to normal medical and surgical management, especially during postoperative rehabilitation [62,63]. A recent randomized controlled study including patients with CRS who underwent FESS found that olfactory training following surgery further enhanced olfactory recovery compared with standard treatment alone, endorsing its application as a straightforward, at-home rehabilitation method [62]. Moreover, an ongoing randomized study is investigating the use of OT for olfactory loss associated with chronic rhinosinusitis, exploring its combination with conventional treatments and identifying possible predictors of improvement [63].

Beyond OT, supportive strategies such as educating patients, providing safety advice (e.g., use of smoke/gas alarms and food safety precautions), and offering psychological support are considered substantial. These strategies help address the major quality-of-life issues associated with smell loss and complement pharmacological and surgical care [5,61,62].

**Figure 2:**
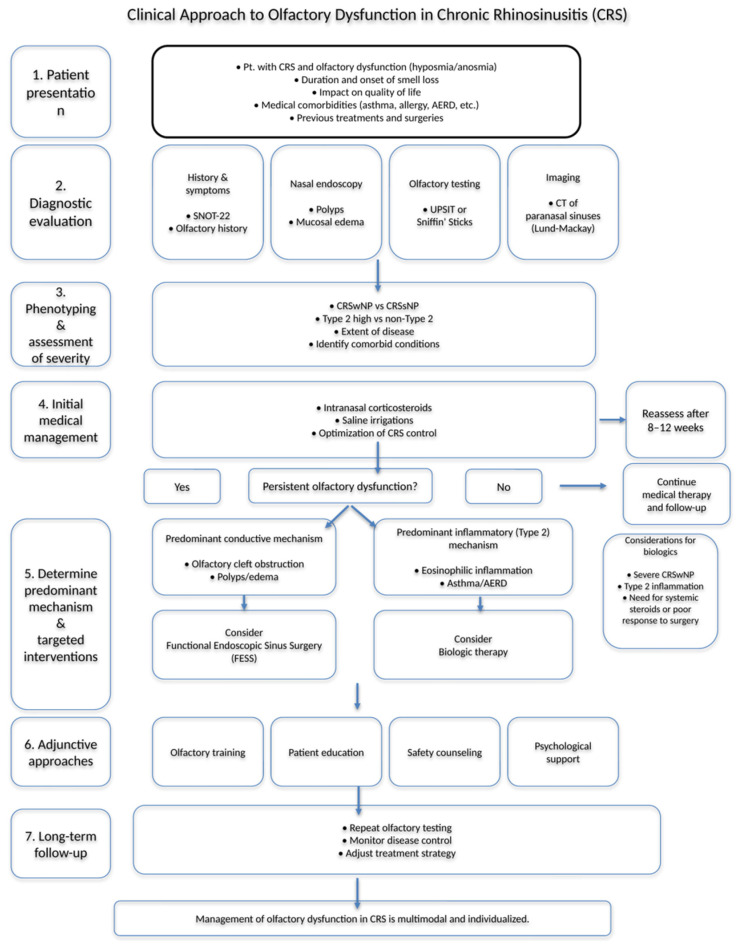
Management algorithm for olfactory dysfunction in chronic rhinosinusitis.

Although OT is inexpensive, safe, and easily available, important doubts remain regarding the optimal training protocol, duration of therapy, and selection of patients most likely to benefit. Consequently, OT should currently be viewed as a complementing component of multimodal olfactory rehabilitation rather than a replacement for established medical or surgical treatment where needed, however more studies are required to substantiate its effectiveness [60,61,62,63] (Figure 2).

## 9. Conclusions

Olfactory dysfunction is a prevalent and clinically meaningful aspect of chronic rhinosinusitis, resulting from a combination of conductive obstruction, inflammation-mediated injury to the olfactory epithelium, disrupted regenerative processes, and secondary changes within central neural pathways. This suggests that olfactory dysfunction in CRS affects not only the paranasal sinuses but the entire olfactory system. Since patients frequently underestimate the severity of their smell problems, an accurate assessment of olfactory dysfunction necessitates a combination of formal psychophysical testing and patient-reported measures. Imaging studies and nasal endoscopy are helpful in detecting structural abnormalities, but are not sufficient in dealing with the underlying sensorineural deficits. Many patients, particularly those with type-2 inflammation, have improved olfactory function with existing treatments, such as intranasal corticosteroids, short courses of oral steroids, Functional Endoscopic Sinus Surgery, and monoclonal antibodies. However, differences in treatment response and recurrence of symptoms emphasize the importance of managing and controlling their chronic inflammation. Careful treatment tailored to their endotypes will be essential in improving treatment responses in patients with CRS-induced olfactory dysfunctions by utilizing more standardized smell function testing and addressing their treatment with more tailored approaches. Olfactory training, which is a type of supportive treatment, has been identified as an important and low-risk treatment approach that may be added to their treatment regimen; however, further studies are needed to confirm its effectiveness.

## Figures and Tables

**Table 1:** Mechanisms of olfactory dysfunction in chronic rhinosinusitis.

Mechanism	Pathophysiology	Clinical Features	Potential Reversibility
Conductive dysfunction	Obstruction of odorant passage to the olfactory epithelium because of nasal polyps, mucosal edema, retained secretions, and olfactory cleft narrowing	Reduced sense of odors, often related to severe nasal blockage and CRSwNP	Often reversible by addressing inflammation and restoring airflow with Medical Therapy or FESS
Inflammation-induced epithelial dysfunction	Chronic type-2 inflammation with elevated eosinophils, IL-4, IL-5, IL-13, IgE and local inflammatory mediators impacting the olfactory mucosa	Olfactory loss disproportionate to the degree of nasal obstruction; often associated with severe eosinophilic CRS	Partially reversible; may improve with corticosteroids, biologics, and treatment of underlying inflammation.
Neuroepithelial injury	Olfactory receptor neuron damage, impaired neurogenesis, epithelial remodeling and neuronal loss secondary to chronic inflammation.	Hyposmia or anosmia that persists despite appropriate sinonasal ventilation	Olfactory recovery is often incomplete, especially in long-standing disease
Central neural alterations	Structural and functional changes in the olfactory bulb and pathways may be the result of chronic deprivation and inflammation.	Persistent olfactory dysfunction despite improvement of sinonasal disease; currently identified mainly through research-based imaging studies	Uncertain; clinical significance and reversibility still remain under investigation
Mixed mechanisms	A combination of conductive obstruction, chronic inflammation, and sensorineural injury	The most common presentation in CRS patients	The outcome depends on the relative contribution of each mechanism and how each responds to treatment.

## Data Availability

No new data were created or analyzed in this study.
